# Análisis comparativo de los centros de detención en España e Italia 2018-2023: Abordar las deficiencias sistémicas y el cumplimiento de los derechos humanos

**DOI:** 10.12688/openreseurope.20108.2

**Published:** 2025-07-14

**Authors:** Laura María Zanón Bayón-Torres, Almudena Juárez Rodriguez, Mercedes Fernández

**Affiliations:** 1 Department of Sociology and Social Work, Comillas Pontifical University Faculty of Human and Social Sciences, Madrid, Community of Madrid, Spain; 2 University Institute of Migration Studies, Comillas Pontifical University Institute of Migration Studies, Madrid, Community of Madrid, Spain

**Keywords:** Detention centres, Internment centres, Spanish Ombudsman, Italian National Guarantor, qualitative content analysis, operational deficiencies, migration control, Fundamental Rights

## Abstract

**Background:**

This article examines the conditions and management of immigration detention centres in Spain and Italy.

**Methods:**

The study analyses the reports of the Spanish Ombudsman and the Italian National Guarantor on visits to detention and internment centres for foreigners to identify similarities and differences in the functioning of these centres depending on the country. Using a qualitative content analysis approach with NVivo 14 software, 16 reports (10 Italian and 6 Spanish) published between 2018 and 2023 were analysed. The reports were selected based on comparability, excluding non-relevant or non-equivalent documents in both countries.

**Results:**

The findings reveal deficiencies in health care, staff training, legal care, hygienic conditions, and transparency in managing removals in both countries. Additionally, there is an abuse of detention as a migration control measure and a de facto use of these centres as first reception centres. A significant difference is the length of detention, which is limited to 60 days in Spain compared to 180 days in Italy.

**Conclusions:**

This analysis highlights the need for reforms to improve conditions in these centres, ensure respect for fundamental rights, and stop their use as first reception centres.

## Plain language summary

This article looks at the conditions and management of immigration detention centers in Spain and Italy. It studies reports from the Spanish Ombudsman and the Italian National Guarantor on visits to these centers to find similarities and differences between the two countries. Using a qualitative content analysis with NVivo 14 software, 16 reports (10 Italian and 6 Spanish) published between 2018 and 2023 were analyzed. The reports were chosen for their comparability, excluding non-relevant or non-equivalent documents.

The results show problems in health care, staff training, legal care, hygiene, and transparency in managing removals in both countries. Additionally, there is an overuse of detention as a migration control measure and these centers are often used as first reception centers. A major difference is the length of detention, which is limited to 60 days in Spain compared to 180 days in Italy. This analysis highlights the need for reforms to improve conditions in these centers, ensure respect for fundamental rights, and stop their use as first reception centers.

## Introduction

Immigrant detention centres are facilities where individuals who are not citizens or legal residents of a country are held while their immigration status is being determined. These centres are used to detain people who are awaiting deportation, those who have violated immigration laws, or those who are seeking asylum. In the European Union, these centres play a key role in internal migration policies, acting as a control measure for foreigners ^1^. They are regulated under Directive 2008/115/EC (Return Directive), which confirms that the centres are designed to ensure the expulsion of persons in an irregular status (persons whose presence in the territory is not regularised in accordance with national immigration law). Article 16 stipulates that detention must be carried out in specialised centres, with a maximum period of six months (Article 15.5) ^2^.

Immigration detention is debated because it conflicts with human rights versus national security and sovereignty ^3^. It constitutes a restriction on liberty ordered by administrative or judicial authorities to facilitate measures such as expulsion ^4^. The Parliamentary Assembly of the Council of Europe noted in 2010 that the detention increase is due to increased arrivals and the tightening of migration policies.

Immigration detention centres in Spain and Italy play a central role in their migration management systems, as both countries serve as primary entry points to Europe. In Spain, these facilities are known as Centros de Internamiento de Extranjeros (CIE), detention centres for foreigners, while in Italy, they are called Centri di Permanenza per il Rimpatrio (CPR), Repatriation Holding Centres.

Legal and political discussions surrounding detention centres have been particularly pronounced in both countries. In Spain, the role of CIE has been the subject of ongoing debate, with incidents involving detainee protests and allegations of inadequate conditions prompting calls for reform. In Italy, some regional governments have expressed reservations about expanding the CPR system, citing concerns about their operation and oversight. Indeed, these centres face various challenges regarding their capacity, legal framework, and human rights considerations.

In Spain, NGOs have documented issues like overcrowding, inadequate medical care, and substandard living conditions in CIE, which are meant to be administrative rather than punitive. This has led to widespread criticism resembling prison environments ^5, 6^. Similarly, in Italy, organisations like the Italian Coalition for Civil Liberties and Rights report ongoing problems, including overcrowding, limited medical services, and poor hygiene conditions ^7^. CPR, designed for administrative purposes, have also been criticised for their prison-like conditions, resulting in protests and legal disputes ^8, 9^.

In addition to their function of custody and detention for foreigners who are to be expelled, the detention centres for foreigners currently perform two other functions. First, they act as first reception centres for foreigners arriving at the European southern border. Second, the excessive use of deprivation of liberty to manage unwanted migration hinders the integration of migrants who remain in the country for prolonged periods. Thus, these centres have become deportation devices and detention centres and are present at all stages of migration control ^10, 11^. In other words, people can be detained in these centres at different stages of their migration process or during their stay in the country. These centres are therefore used at the border upon arrival, where they serve as places of identification and initial detention, with the deprivation of liberty being abused. Secondly, they are used as a precautionary measure while awaiting the resolution of the expulsion order, with admission possible after a more or less prolonged stay in the country and even after the occurrence of a subsequent administrative irregularity. Finally, once the expulsion order has been resolved, they function as detention facilities until the return measure is enforced.

The recent approval of the New European Pact on Migration and Asylum in December 2023 introduces changes to the EU's approach to migration governance ^12^. The pact aims to streamline border procedures, differentiate between asylum seekers and economic migrants, and reinforce mechanisms for returning individuals without legal residence status ^13^. It also establishes solidarity among EU member states, allowing them to contribute by relocating asylum seekers or providing financial support to countries managing significant arrivals ^14^, such as Spain and Italy. While these provisions seek to create a more coordinated European migration system, they have also prompted discussion regarding their potential impact on detention policies -including provisions for expanding screening and return procedures- and raised concerns among human rights organisations and scholars about the balance between efficient processing and the rights and freedoms of migrants ^15^.

The paper's main aim is to compare and contrast the functioning of detention and internment centres for foreigners in Spain and Italy by analysing reports from the Spanish Ombudsman and the Italian Garante Nazionale. The study seeks to identify similarities and differences in the conditions and management of these centres ^16^, highlighting deficiencies and the need for reforms to improve conditions and respect for fundamental rights. These two countries were selected due to their geostrategic position and the concern raised by these centres. Furthermore, the project within which this article is framed, and in which both countries are partners, justifies this comparison.

## The Spanish CIE and the Italian CPR: a comparative overview

Currently, in Italy, there are nine CPRs in operation, distributed in seven regions: Bari and Brindisi (Puglia), Caltanissetta and Trapani (Sicily), Gradisca (Friuli-Venezia Giulia), Macomer (Sardinia), Milan (Lombardy), Rome (Lazio, Ponte Galeria, the only one with a section for women) and Palazzo San Gervasio (Basilicata), In the case of Spain, there are seven CIE, distributed in six regions: Aluche (Comunidad Madrid), La Piñera (Algeciras-Andalusia), Zapadores (C. Valenciana), Sangonera La Verde (Murcia), Barranco Seco (Las Palmas), Matorra (Fuerteventura) both in the Canary Islands and Zona Franca (Barcelona-Catalonia).

According to the statistics provided by the Italian National Guarantor ^17^, the Servicio Jesuita Migrantes ^18, 19^ and the Spanish Ombudsman ^20^, (the most recent comparative statistics available), there were 6,383 and 2,276 people detained in Italian CPRs and Spanish CIE respectively. The main nationalities of inmates were Morocco and Senegal, which are in Spain, Egypt, and Tunisia, which are in Italy. The average stay is 30.2 days for Spain and 39.8 for Italy. The two leading causes of leaving a CIE are practical repatriation and release from detention. For both countries, the effective repatriations amount to slightly more than 50% of the total inmates and have remained constant over the years. According to the Spanish Ombudsman (2023), the percentage of people expelled from these centres was 36.5%, while in Italy it fluctuates between 45% and 50% ^17^.

The following Table 1 summarises the comparative view of both institutions.

**Table 1.** Functioning of Spanish CIE and Italian CPR.

Aspect	CIE	CPR
**Governing Laws**	Organic Law 4/2000 Royal Decree 162/2014	Legislative Decree 286/1998 Legislative Decree 142/2015 Law 173/2020 Decree Law 130/2020 Interior Ministry Directive of 19 May 2022
**Max Detention** ** Period**	60 days	90 days, extendable to 180 days
**Rights of Detainees**	Healthcare Legal aid Communication with the outside world	
**Challenges**	Overcrowding inadequate medical care substandard living conditions	
**Oversight ** **Mechanisms**	Juez de Control (magistrate with specific powers to supervise detention conditions in CIEs and ensure respect for the fundamental rights of detainees) Spanish Ombudsman	National Guarantor Ministry of Interior
**Reforms**	Better living conditions Transparency detainee rights protection detention times reduction	Simplified procedures for the design of reception, stay and repatriation facilities.

Source: own elaboration.

In Spain, Organic Law 4/2000 on the Rights and Liberties of Foreigners ^21^ and its amendments govern the regulation of CIE. Royal Decree 162/2014 ^22^ provides additional regulations regarding these centres' organisation, management, and conditions. In Italy, Legislative Decree No. 286/1998, known as the Testo Unico sull'Immigrazione ^23^, Legislative Decree 142/2015, and Law 173/2020 (plus Decree Law 130/2020) ^24– 26^ regulate CPR, the rights of detainees, detention conditions, and the standards of facilities. The Interior Ministry Directive of 19 May 2022 elaborates on their operational guidelines and management standards ^27^.

The maximum legal detention period is 60 days for Spain, while in Italy, it is 90 days, extendable to 180 days. Both periods are shorter than the 6-month limit established in the Return Directive, which can be extended to 12 months in exceptional cases (Article 15, Return Directive). Both Spanish CIE and Italian CPR temporarily detain foreigners in an irregular status to ensure their identification and expulsion, restricting their freedom of movement.

Both countries guarantee rights such as communication with the outside world, legal, health and social care, and comprehensible information about their situation. Both systems provide for exceptions for vulnerable groups, such as minors, victims of trafficking and pregnant women. Social assistance is regulated in Art. 11 of Legislative Decree No. 142/2015 ^24^ in Italy and Art. 15 of Royal Decree 162/2014 ^22^ in Spain, ensuring minimum food, health and hygiene standards.

Oversight mechanisms for CIE in Spain involve judicial authorities, especially the Judge of Control, who monitors conditions and ensures detainee rights are upheld (Royal Decree 162/2014 art. 62.6). In addition, the Spanish Ombudsman, designated as the National Preventive Mechanism ^28^, inspects these facilities to prevent torture and inhumane treatment and produces annual reports detailing findings from CIE inspections and recommendations ^20, 29^. As for Italy, the monitoring is carried out by the National Guarantor for the Rights of Persons Detained or Deprived of Liberty, which inspects facilities and ensures detainees' rights are protected ^8^. The Ministry of Interior oversees the overall system. For both countries, NGOs provide independent reports and advocacy frameworks ^18, 30^. Unlike what may happen in other countries, these centres are managed directly by the Ministry of the Interior in both countries, although they do involve the participation of the third sector in the provision of various services such as social care.

In recent years, Spain's CIE and Italy's CPR systems have faced calls for reform. Advocacy from the Spanish Ombudsman and human rights organisations has led to Spanish government reforms for better living conditions, transparency, detainee rights protection, and detention times reduction ^31, 32^. In the same vein, Italian CPR have incorporated the improvements requested by the National Guarantor ^17^; furthermore, Italy has experienced legislative changes to simplify and implement procedures for the design of reception, stay and repatriation facilities ^33^. However, despite these efforts, effective implementation remains a challenge and conditions in many CPR continue to be criticised by human rights organisations ^34^.

## Methodology

The reports published by the Spanish Ombudsman and the Italian National Guarantor cover the visits made by both institutions to the internment and detention centres for foreigners. These reports reveal shortcomings, deficiencies, needs for improvement, violations of human rights and proposals for improvement, and the progress made in implementing the recommendations of both institutions.

A qualitative methodological approach with content analysis has been used. A sample of 23 reports published by the Italian National Guarantor and eight by the Spanish Ombudsman were collected between 2018 and 2023. Following a selection process explained below, the analysis was conducted on 10 Italian and 6 Spanish reports from 2018 and 2023. A purely descriptive content analysis was carried out, discarding a semiotic and discourse analysis ^35, 36^ because it was necessary to translate the reports and, therefore, the singularities of each language may have been lost or blurred. With regard to the Italian reports, an automatic translation tool (specifically DeepL in its paid version) was used to facilitate their analysis in Spanish. Although this resource allows access to the general content of the documents, it is recognised that it may entail some loss of linguistic nuances and terminological specificity.

The selection process was based on determining which reports could be compared between the two countries. The Italian National Guarantor publishes notes and communications to different police and political agents that have not been included in the analysis for two reasons. Firstly, it often does not provide information on the CPR, and secondly, there are no equivalent publications in the Spanish Ombudsman. Therefore, only those reports published between 2018 and 2023 that reflect the visits and analyses of both ombudsmen on the state and the functioning of the centres have been analysed, regardless of the length of these reports. This resulted in a total of 16 reports. Although the classification of the reports could be based on various criteria such as date, type of centre, population admitted, date, place, etc., to compare countries, it was decided that the classification should be by publication date and country. The difference in the reporting practices of the Spanish and Italian Ombudsmen has meant that there are more Italian sources available, although this does not mean that there is more information about Italy than about Spain. On the contrary, we consider that the information is similar, since one of the main differences is that the Spanish Ombudsman publishes a single report covering all visits to the different centres, while the Italian Ombudsman publishes separate reports on the different centres.

After reading the reports, a comparison was made between the headings and the topics addressed to identify those issues that could be compared. We then conducted a content analysis of these reports using qualitative analytical strategies in the Nvivo 14 programme, where primary and secondary coding structures were established ( Table 2). The analysis section was organised into three key thematic blocks based on these.

**Table 2.** Code and description.

Codes	Description	
	SPAIN	ITALY
**Structural and operational conditions**		
Use of rooms and facilities	Deteriorated and poorly maintained facilities (Valencia, Murcia, Madrid). Inappropriate use of space (consultation rooms and isolation). Violations of EU regulations (mixing of profiles and privacy).	Unsanitary conditions and inadequate furniture. Serious hygiene problems (insects and rodents). Overcrowding and prolonged detention (90 to 180 days).
Structural deficits	Structural defects: emergency doors, mould and heating.	Structural deterioration and lack of privacy. Centres with prison-like design.
**Detainee rights**		
Comunication	Restrictions on mobile phone use and physical communication (screens in visiting rooms). Unequal access to public telephones depending on the centre.	Severe restrictions on mobile phone use (2022–2023). Structural difficulties in communicating from cells.
Information	Late notification of expulsions; lack of clear and accessible information on procedures.	Lack of transparency in detention and repatriation processes. Delays in asylum procedures.
Complaints and claims	Absence of standardised reporting protocols until 2020–2021; subsequent partial improvements without addressing the lack of effective channels.	Absence of accessible complaint mechanisms. Invisibility of complaints in contexts of prolonged detention.
Visits and relations with the outside world	Physical restrictions (partitions) Recent minor structural improvements (openings in partitions). Limited contact with family members.	Restricted visiting hours for lawyers. Limited possibility of external contact, especially in pre- deportation detention.
**The adequacy of healthcare, legal,** **and social support services**		
Legal assistance	Legal assistance only available at certain centres (Madrid, Barcelona, Valencia). Lack of privacy during legal consultations (2020–2023).	Irregular access to lawyers (2018–2019). Discontinuity in legal representation (2023). Delays in processing asylum applications.
Health care	Delays in mandatory medical examinations. Lack of permanent medical staff (except in Madrid). Lack of mental health care. Language barriers without professional interpreters. Poor medical records (2022–2023).	Insufficiently trained medical staff. Language and cultural barriers. Excessive use of psychotropic drugs and lack of suicide prevention protocols. Long waiting times for psychiatric care (Milan, Turin).
Social services	Very limited or no social services. Lack of educational or recreational programmes.	Social support programmes are virtually non-existent. There are no activities to mitigate the psychological impact of detention.

Source: own elaboration.

The analysis involved coding the text from the reports according to these predefined categories. Each report was meticulously examined to extract relevant information that fit into the established codes. This process allowed for a comprehensive comparison of the conditions and management practices in detention centres across Spain and Italy. After an initial exploratory reading of the reports, a primary and secondary coding structure was agreed upon and implemented in the NVivo 14 programme. During the analysis process, regular meetings were held to review the application of the codes and resolve any discrepancies through discussion and agreement. This strategy ensured consistency in the thematic interpretation of the reports and provided methodological rigour to the qualitative analysis, in line with recommendations for comparative studies using documentary sources ^37^. The data collection and analysis were conducted from June to November 2024. The findings were then organized into three thematic blocks, which provided a structured framework for discussing the results. This approach ensured that the analysis was thorough and that all relevant aspects of the detention centres were considered.

## Results: comparative analysis of foreign detention centers in Spain and Italy (2018–2023)

This section offers a comparative analysis of the reports by the Spanish Ombudsman and the Italian National Guarantor on foreign detention centres— *CIE and CPR*—from 2018 to 2023. As established in the Methods section, the analysis focuses on three key dimensions: structural and operational conditions, detainee rights, and the adequacy of healthcare, legal, and social support services.

### Structural and operational conditions

Detention centres in both countries face significant operational and structural deficiencies. In Spain, many centres suffer from inadequate facilities and poor maintenance. Reports from 2018 and 2019 highlighted issues such as the multifunctional use of spaces for medical consultations, suicide prevention, and disciplinary segregation. These challenges were particularly evident in Valencia and Murcia, where surveillance systems were outdated, and bathroom facilities were deteriorated.

By 2020 and 2021, violations of European Union norms became evident, as asylum seekers were often housed alongside other detainees. Madrid's CIE faced scrutiny for poor record-keeping and excessive police presence. Reports from 2022 and 2023 documented ongoing problems, including malfunctioning emergency doors, privacy breaches during medical consultations, and structural issues such as water leaks, mould, and heating system failures.

Italy faced similar challenges, with reports from 2018 and 2019 describing unsanitary conditions, including broken windows, poor lighting, and inadequate furnishings. In some centres, such as Bari, detainees were compelled to eat on the floor due to the absence of tables and chairs. Infestations of insects and rodents compounded hygiene problems.

From 2020 to 2023, overcrowding and a heightened securitisation of detention centres aggravated the situation. Extending detention periods from 90 to 180 days further strained conditions, increasing tensions among detainees. Reports highlighted worsening structural conditions, particularly in Bari and Turin, where privacy violations, water leaks, and mould were common. The prison-like design of many facilities reinforced the punitive nature of detention.

### Detainee rights

The protection of detainees' rights emerged as a critical issue in both countries, with systemic barriers impeding communication, access to legal counsel, and procedural fairness.

In Spain, reports from 2018 and 2019 noted significant communication restrictions. Visiting rooms often featured glass partitions, preventing physical contact, while access to mobile phones varied across centres. During 2020 and 2021, inadequate notification periods for deportations and the absence of standardised complaint protocols exposed detainees to systemic vulnerabilities. Reports from 2022 and 2023 noted minor improvements, such as small openings in partitions, but communication barriers and disparities in access to public telephones persisted.

In Italy, detainees faced limited access to legal representation and delayed asylum claim processing. Reports from 2018 and 2019 described restricted visiting hours for lawyers and a lack of transparency in pre-deportation holding cells. By 2020 and 2021, administrative detention was criticised as an inefficient migration control tool, with only around half of detainees ultimately repatriated. From 2022 to 2023, severe restrictions on mobile phone use and delays in asylum processing, particularly in Turin and Milan, left detainees vulnerable to legal uncertainties.

### Healthcare, legal, and social support services

Healthcare services in detention centres were deeply inadequate, with systemic issues affecting both countries.

The 2018 and 2019 reports in Spain highlighted delays in mandatory medical examinations and inconsistent medication management during detainee transfers. By 2020 and 2021, the absence of permanent medical staff in most centres—except Madrid—was noted, alongside a lack of mental health support. Language barriers further hindered access to care, with detainees relying on peers for translation. Reports from 2022 and 2023 underscored the absence of professional interpreters, poor record-keeping, and inadequate documentation of medical treatments.

In Italy, healthcare challenges included insufficiently trained medical staff, cultural and linguistic barriers, and poor management of mental health crises. Reports from 2018 and 2019 noted the misuse of psychotropic medications and the absence of protocols for managing suicidal behaviour. By 2020 and 2023, long waiting times for psychiatric evaluations and systemic overprescription of psychotropic drugs were significant concerns, particularly in Milan and Turin.

Legal and social support services were fragmented and inconsistent in both countries. Legal assistance was available in only a few centres in Spain, such as Madrid, Barcelona, and Valencia. From 2020 to 2023, the lack of privacy during legal consultations undermined detainees' access to justice.

In Italy, similar deficiencies persisted. Reports from 2018 and 2019 highlighted delays in asylum claim processing and inconsistent access to lawyers. By 2023, detainees often experienced discontinuity in legal representation, as they were assigned different lawyers throughout their detention. Social support services, including recreational and educational programs, were almost absent in both countries, exacerbating the psychological impact of detention.

## Discussion

The comparative analysis of detention centres in Spain and Italy reveals systemic deficiencies undermining detainees' rights and well-being. Both countries face similar challenges, including structural decay, unsanitary conditions, and inadequate healthcare and legal services. However, the situation in Italy is particularly critical due to more pronounced securitisation, pervasive healthcare deficiencies, and systemic communication barriers. This discussion contextualises the observed deficiencies, highlighting their impacts and the influence of migration policy securitisation and recommending reforms to align practices with international human rights standards.

### Structural and operational deficiencies

Spain and Italy face serious structural and operational deficiencies within their detention systems. In Spain, centres suffer from infrastructural decay, including broken emergency doors, malfunctioning heating systems, and unsanitary conditions. These deficiencies compromise safety and exacerbate detainees' physical and mental distress ^38^. Italy's *CDRs* similarly struggle with overcrowding, poor hygiene, and a lack of privacy in sanitary spaces ^39^.

Research consistently highlights the harmful effects of substandard detention infrastructure. Poor living conditions, prison-like architecture, and overcrowding contribute to detainees' sense of isolation and stigmatisation, reinforcing perceptions of detention as punitive rather than administrative ^40^. For instance, medical consultation rooms are used as segregation cells in Spain. This breaches privacy and safety standards, going against international guidelines like the Nelson Mandela Rules ^41, 42^.

Italy's detention centres reflect an even more securitised approach, where rigid, penal designs amplify detainees' psychological harm. Studies show that securitised facilities intensify feelings of alienation and diminish overall well-being ^43^. The persistent lack of infrastructural investment in both systems perpetuate systemic neglect, raising ethical and legal concerns about the treatment of detained individuals.

### The health impacts of detention

The adverse health impacts of immigration detention are well-documented, with evidence showing that prolonged detention exacerbates physical and mental health issues, particularly among vulnerable groups such as asylum seekers. Prolonged confinement has been linked to anxiety, depression, and post-traumatic stress disorder ^44^.

Italy's CPR illustrate a medicalised but insufficient response to mental health crises. An overreliance on sedatives, often prescribed without adequate oversight, reflects a tendency to address symptoms rather than underlying causes ^45, 46^. This approach underscores the lack of trained professionals capable of providing appropriate psychiatric care and highlights systemic neglect of detainees' mental health.

Similarly, detention centres in Spain face delays in medical examinations, insufficient psychiatric care, and reliance on detainees to translate for one another due to a lack of professional interpreters. Research suggests that linguistic and cultural barriers in healthcare delivery exacerbate misdiagnoses and delays in treatment, compounding health inequities ^47^. These deficiencies undermine detainees' right to adequate healthcare and perpetuate systemic vulnerabilities.

### Legal and procedural deficiencies

Access to legal representation and procedural safeguards is critical for protecting detainees' fundamental rights. However, both Spain and Italy exhibit systemic failures in this area. Limited access to legal counsel, inconsistent deportation notifications, and delays in asylum processing expose detainees to prolonged uncertainty and, in some cases, indefinite detention.

In Italy, frequent legal representation changes disrupt asylum seekers' defence strategies. Research shows that such procedural inefficiencies exacerbate detainees' psychological distress and undermine their trust in the legal system ^48, 49^. Similarly, Spain's inconsistent deportation processes raise concerns about transparency and accountability, reflecting broader critiques of administrative detention as an opaque and punitive tool ^43^.

These legal deficiencies create a "legal limbo" for detainees, heightening their vulnerability and eroding procedural fairness. This lack of clarity and continuity contributes to detainees' isolation and distrust of legal and institutional frameworks ^50^.

### Securitisation of migration policies

The observed challenges in Spain and Italy reflect a broader European trend towards securitising migration policies. This approach frames migration as a security threat, legitimising punitive measures such as detention and deportation ^51^. Italy's extension of detention periods to 180 days exemplifies this securitised approach, prioritising deterrence over humane treatment.

Recent research questions the effectiveness of detention as a tool for migration control. Aiken & Silverman ^52^ demonstrate that prolonged detention does not significantly reduce irregular migration or increase deportation rates. Instead, many detainees are ultimately released, raising ethical questions about the proportionality and necessity of detention.

The securitisation of migration also reinforces social stigmatisation, portraying migrants as threats rather than individuals entitled to protection and dignity. The prison-like design of Italian CPR reflects this punitive framing, compounding detainees' psychological distress and alienation ^50^. Addressing these issues requires a shift towards policies that respect human dignity and adopt rights-based alternatives to detention ^53^.

### Recommendations

Comprehensive reforms guided by scientific evidence and aligned with international human rights standards are essential to address the systemic deficiencies in Spanish and Italian detention centres.

Under operational issues, urgent renovations are needed to improve hygiene, safety, and living conditions, ensuring compliance with international standards. Additionally, trained professionals should provide comprehensive healthcare services, including specialised mental health care, with oversight to prevent overmedication.

Control mechanisms should include standardised access to confidential legal representation, timely deportation notifications, and clear asylum processing protocols, which are crucial for upholding detainees' rights. Regular inspections by independent monitoring bodies are also essential to ensure detention centres meet human rights standards and to hold them accountable for systemic failures.

As an alternative approach, research supports non-custodial options such as community-based housing and case management programs, which are more humane and cost-effective than detention.

## Conclusion

The systemic deficiencies in Spanish and Italian detention centres—ranging from infrastructural decay to failures in healthcare and legal protections—reflect broader challenges within European migration governance. The scientific literature confirms that detention exacerbates mental health issues, undermines procedural rights, and fails to achieve its stated objectives of controlling migration.

The analysis carried out focuses on a specific period, which has not allowed for an in-depth examination of how the new European Union Pact on Migration and Asylum of 2023 will affect the situation. We therefore consider it necessary for future research to take this aspect into account. Tackling these challenges necessitates a fundamental shift to a rights-based approach emphasising humane treatment, transparency, and accountability. Non-custodial alternatives to detention and targeted investment in infrastructure, healthcare, and legal safeguards offer a more ethical and practical pathway forward. Aligning detention practices with international human rights standards is essential to safeguarding the dignity and well-being of all detainees, ensuring that migration governance is grounded in principles of justice and humanity.

## Ethics and consent

Ethical approval and consent were not required.

## Data Availability

The data supporting the findings of this study have been extracted from the reports of the Spanish Ombudsman and the Italian Ombudsman. These reports are publicly accessible on the respective websites of these institutions. The links to the reports are in https://doi.org/10.5281/zenodo.15254336 Zenodo : Comparative analysis of detention centres in Spain and Italy 2018–2023: Addressing systemic deficiencies and human rights compliance https://doi.org/10.5281/zenodo.15254336 ^54^ Data are available under the terms of the Creative Commons Attribution 4.0 International license (CC-BY 4.0).

## Software availability

This research utilized NVivo (QSR International), a proprietary software for qualitative analysis. No supplementary software was developed or employed; therefore, no source code is available.
