# Supplementary material for: Análisis comparativo de los centros de detención en España e Italia 2018-2023: Abordar las deficiencias sistémicas y el cumplimiento de los derechos humanos
Source: Open Res Eur. 2025 Jul 14;5:124. Originally published 2025 May 8. [Version 2] doi: 10.12688/openreseurope.20108.2 (PMC12238845; doi:10.12688/openreseurope.20108.2)
Supplement: Peer review history

# Reviewer report: Reviewer response for version 2

**DOI:** 10.21956/openreseurope.22585.r54538

**Published:** 2025-08-23

**Authors:** Francesca Esposito

**Affiliations:** 1 University of Bologna, Cesena, Cesena, Italy

The article provides a comparative analysis of conditions in Spanish and Italian immigration detention centres, based on reports by the Spanish Ombudsman and the Italian National Guarantor covering the period 2018–2023. Given the increasing reliance on detention as a tool to manage migration flows across European states, and in view of the forthcoming implementation of the New European Pact on Migration and Asylum in 2026, it is crucial for scholars across disciplines to engage in a critical examination of the detention system.

 Although the study is relevant and timely, and it highlights important criticisms emerging from the comparative analysis as well as providing valuable recommendations, it also contains several aspects that require further improvement before it can be considered for publication. Below, I provide feedback for each section. My comments primarily focus on the Italian part of the analysis, as this falls within my area of expertise.

**Introduction**

 The Introduction is well structured and addresses key points in order to introduce the reader to immigration detention and highlight key aspects of the Spanish and Italian detention systems. It is explained how immigration detention centres serve different functions and how the people who are confined within them have different backgrounds and situations.

 Regarding the presentation of the Italian detention system, the authors address some important points and substantiate their claims with relevant sources. However, significant literature—particularly grey literature produced by civil society organisations—is missing. For instance, while they cite a short online article by the Italian Coalition for Civil Liberties and Rights (CILD), they omit several important reports produced by CILD over the years. Similarly, they do not engage with key reports from other national and international groups and civil society organisations, such as Border Criminologies, ASGI (Associazione Studi Giuridici sull’Immigrazione) and ActionAid, just to mention a few. This lack of engagement with a rich body of grey literature represents a limitation that should be addressed.

 here is also a specific section of the Introduction that is particularly unclear to me. In the fifth paragraph, the authors discuss the functions of immigration detention—a complex issue extensively debated by critical scholars in this field (see, for instance, Brandariz, Fabini, Fernández-Bessa & Ferraris, 2025, *Border Criminologies from the Periphery*)—and state:

*“In addition to their function of custody and detention for foreigners who are to be expelled, the detention centres for foreigners currently perform two other functions. First, they act as first reception centres for foreigners arriving at the European southern border. Second, the excessive use of deprivation of liberty to manage unwanted migration hinders the integration of migrants who remain in the country for prolonged periods. Thus, these centres have become deportation devices and detention centres and are present at all stages of migration control.”*

 I would encourage the authors to clarify and expand on this statement. Specifically, it is unclear how pre-removal detention centres, such as CPRs, can act as *“reception centres”* for newly arrived foreign nationals. Furthermore, do the authors mean that one of the functions of immigration detention centres is to hinder migrants’ integration? If so, for what purpose? Overall, this paragraph would benefit from revision for enhanced clarity, and I believe it is crucial to clearly differentiate between detention and reception, or else to explain how these two functions specifically overlap.

 Finally, it is unclear why the authors distinguish between internment centres and detention centres for foreigners. Regardless of the official euphemisms used to designate them, in both countries these centres operates as immigration detention centres.

**The Spanish CIE and the Italian CPR: a comparative overview**

 This section of the article introduces the immigration detention systems in Spain and Italy, outlining their main characteristics. However, with regard to the Italian detention system, there are some inaccuracies and instances of outdated information.

 Firstly, since October 2023, the maximum detention period has been reverted to the pre-2014 duration of 18 months (Law Decree 19 September 2023, n. 124); it is therefore no longer six months. Secondly, the system currently comprises ten immigration detention centres (CPRs) within the national territory, as the Turin CPR was reopened in March 2025. Furthermore, an eleventh detention centre was established in late 2024 in Gjadër, Albania, under the framework of the recent Italy–Albania protocol. This latter development is particularly significant, as many researchers have highlighted, because it represents an unprecedented shift in the Italian immigration detention paradigm.

 I would also encourage the authors to conduct a thorough revision of the legislative frameworks governing immigration detention in Italy, as several legislative reforms have been introduced in recent years. Moreover, I suggest emphasising that the main legal framework governing immigration detention in Italy is the Legislative Decree 286/1998 **, **which originally established the Italian detention system, with many subsequent provisions serving as amendments or complements to this law. It is also important to highlight that, although Italy’s detention centres formally fall under the authority of the territorially competent Prefettura –UTG, a local branch of the Ministry of Interior that oversees the centres, their management and support services are delegated to private actors, primarily third-sector organisations.

 Finally, I find the reference to Law 162/2023 as a reform incorporating the recommendations of the Italian National Guarantor particularly unclear, or rather incorrect. On the contrary, the National Guarantor published a highly critical analysis of this legislative development. You can find it here: ( refer to 1 )

**Methodology**

 My main criticism and concern relates to the authors’ reliance on an automatic translation tool (DeepL, paid version) to interpret and analyse the Italian reports. This approach risks losing important nuances and meanings—a limitation partly acknowledged by the authors themselves (see p. 6). Such reliance may cause inaccuracies in the analysis of the Italian detention context and conditions, such as those highlighted in previous sections.

 Additionally, I think it would be important for the authors to explain why they have decided to focus on reports published between 2018 and 2023, and do not include in their analysis more recent ones. Indeed, as mentioned above, some significant changes have taken place recently, e.g., regarding the maximum detention period, which are not reflected in the study findings.

 Finally, it is unclear to me what “irregular access to lawyers (2018-2019)” refers to in Table 2 (page 7). I would appreciate if authors can clarify this point.

**Findings**

 I find the presentation of the findings well organized. However, in some instances, the information appears overly condensed, and providing additional details would help enrich the reader’s understanding of the points raised.

**Discussion**

 The discussion is interesting and raises important points. However, I think this section would benefit for further engagement with existing literature, particularly studies and reports focused on the Spanish and Italian immigration detention contexts.

 Furthermore, the discussion should be revised to reflect more recent legislative developments and changes in the detention regime (e.g., length).

**Recommendations**

 Although I agree that the system requires significant changes if we want to uphold migrant people’s fundamental rights and dignity, I have concerns about the proposed intensification of psychiatric care in detention as a strategy to achieve this goal and to “address the underlying causes” of detained people’s suffering. Numerous studies have shown that such suffering is intrinsically linked to the deprivation of liberty itself. I therefore encourage the authors to reflect critically on this point, and more broadly on the assumptions and potential unintended consequences of their recommendations—particularly in terms of how such measures may (or may not) contribute to safeguarding migrants’ dignity and human rights. In this respect, the authors may wish to engage with the growing body of literature addressing the side effects of attempts to “humanise” detention centres.

 Furthermore, I would encourage the authors to further elaborate on their recommendation for **c**ommunity-based alternatives, especially since their own analysis portrays a system that is inherently flawed and structurally causes suffering.

**References**

 Please note that ASGI is Associazione (with only one “z”)

If applicable, is the statistical analysis and its interpretation appropriate?

Not applicable

Is the study design appropriate and is the work technically sound?

Partly

Is the work clearly and accurately presented and does it engage with the current literature?

Partly

Are the conclusions drawn adequately supported by the results?

Partly

Are sufficient details of methods and analysis provided to allow replication by others?

Yes

Are all the source data and materials underlying the results available?

Yes

Reviewer Expertise:

immigration detention in Italy; intersectionality; community-based participatory research; community health promotion.

I confirm that I have read this submission and believe that I have an appropriate level of expertise to confirm that it is of an acceptable scientific standard, however I have significant reservations, as outlined above.

## References

[1] https://www.garantenazionaleprivatiliberta.it/gnpl/page/it/parere_del_garante_nazionale_sul_decretolegge_19_settembre_2023_n_124_recante_disposizioni_urgenti_in_materia_di_politiche_di_coesione_per_il_rilancio_delleconomia_nelle_aree_del_mezzogiorno_del_paese_nonche_in_materia_di_immigrazione?contentId.

# Reviewer report: Reviewer response for version 2

**DOI:** 10.21956/openreseurope.22585.r56710

**Published:** 2025-08-20

**Authors:** Virginia Paloma

**Affiliations:** 1 Universidad de Sevilla, Seville, Seville, Spain

I have no further comments to make.

If applicable, is the statistical analysis and its interpretation appropriate?

Not applicable

Is the study design appropriate and is the work technically sound?

Yes

Is the work clearly and accurately presented and does it engage with the current literature?

Yes

Are the conclusions drawn adequately supported by the results?

Yes

Are sufficient details of methods and analysis provided to allow replication by others?

Partly

Are all the source data and materials underlying the results available?

Yes

Reviewer Expertise:

community participation, mental health, migration, social justice, well-being.

I confirm that I have read this submission and believe that I have an appropriate level of expertise to confirm that it is of an acceptable scientific standard.

# Reviewer report: Reviewer response for version 2

**DOI:** 10.21956/openreseurope.22585.r56712

**Published:** 2025-08-20

**Authors:** M. Alvi Syahrin

**Affiliations:** 1 Immigration Polytechnic University in Depok, Jawa Barat, Jawa Barat, Indonesia

Upon careful reading and evaluation of the manuscript "Análisis comparativo de los centros de detención en España e Italia 2018-2023: Abordar las deficiencias sistémicas y el cumplimiento de los derechos humanos",  I am pleased to **recommend for indexing**. This article makes a significant contribution to the field and meets the high-quality criteria we expect from submissions.

If applicable, is the statistical analysis and its interpretation appropriate?

Yes

Is the study design appropriate and is the work technically sound?

Yes

Is the work clearly and accurately presented and does it engage with the current literature?

Yes

Are the conclusions drawn adequately supported by the results?

Yes

Are sufficient details of methods and analysis provided to allow replication by others?

Yes

Are all the source data and materials underlying the results available?

Yes

Reviewer Expertise:

migration, refugees, detention

I confirm that I have read this submission and believe that I have an appropriate level of expertise to confirm that it is of an acceptable scientific standard.

# Reviewer report: Reviewer response for version 1

**DOI:** 10.21956/openreseurope.21752.r54541

**Published:** 2025-07-08

**Authors:** Virginia Paloma

**Affiliations:** 1 Universidad de Sevilla, Seville, Seville, Spain

The manuscript presents a comparative analysis of the functioning of immigrant detention centers in Spain and Italy. This analysis is based on a qualitative content analysis of 16 reports published between 2018 and 2023 by the Spanish Ombudsman and the Italian National Guarantor. In this way, the manuscript offers a valuable snapshot of the current state of detention centers in both countries.

 The findings suggest that detention centers maintain prison-like conditions and deficiencies in health care (particularly a lack of mental health support), professional interpreters, recreational and educational programs, staff training, legal care, hygienic conditions, and transparency in managing removals in both countries.

 Although the authors make important recommendations based on their findings, I would have liked them to elaborate which elements the New European Pact on Migration and Asylum should specifically consider in order to guarantee human rights in border management. In addition, I believe that the discussion of the results obtained can benefit from the use of a human rights conceptual framework, such as the FREDA principles, which focus specifically on five human rights that social providers should promote (i.e., fairness, respect, equality, dignity, autonomy; see, for example, Curtice & Exworthy, 2010; Health Information and Quality Authority, 2019).

 Furthermore, the authors do not explain why they limited their focus to reports published between 2018 and 2023. On the other hand, the authors state that it was necessary to translate the Italian reports into Spanish to proceed with their analysis. More information on the procedure followed is needed to ensure the reports were properly translated and analyzed.

 As the comparative analysis is based solely on reports published by the Spanish Ombudsman and the Italian National Guarantor, the study may reflect a bias toward deficiencies and areas needing improvement in detention centers. In this sense, I believe the authors should acknowledge this limitation and, in future studies, consider the perspectives from the staff working in these centers and the detainees themselves. This would enable us to analyze the situation from different perspectives and validate the results.

 Finally, I suggest avoiding terminology that does not ensure inclusive equity in language or that reinforces stigma and is deficit-based (e.g., “vulnerable groups”; for alternatives, see National Collaborating Centre for Determinants of Health, 2023).

 In any case, the results clearly show how both detention systems perpetuate the structural violence of border control established by the receiving countries in the Global North against people affected by global inequities. Moreover, the manuscript reflects the crucial role of public institutions and NGOs in promoting human rights and provides evidence-based support for systemic change that offers dignified, rights-based alternatives to detention.

If applicable, is the statistical analysis and its interpretation appropriate?

Not applicable

Is the study design appropriate and is the work technically sound?

Yes

Is the work clearly and accurately presented and does it engage with the current literature?

Yes

Are the conclusions drawn adequately supported by the results?

Yes

Are sufficient details of methods and analysis provided to allow replication by others?

Partly

Are all the source data and materials underlying the results available?

Yes

Reviewer Expertise:

community participation, mental health, migration, social justice, well-being.

I confirm that I have read this submission and believe that I have an appropriate level of expertise to confirm that it is of an acceptable scientific standard, however I have significant reservations, as outlined above.

## References

[1] : FREDA: a human rights-based approach to healthcare. *The Psychiatrist* .2010;34(4) : 10.1192/pb.bp.108.024083 150-156 10.1192/pb.bp.108.024083

[2] Health Information and Quality Authority (2019). Guidance on a human rights-based approach in health and social care services. Irish Human Rights and Equality Commission. https://www.hiqa.ie/sites/default/files/2019-11/Human-Rights-Based-Approach-Guide.PDF.

[3] National Collaborating Centre for Determinants of Health (2023). Let’s Talk: Language of health equity. Antigonish, NS: NCCDH, St. Francis Xavier University. https://nccdh.ca/resources/entry/lets-talk-language-of-health-equity.

# Reviewer report: Reviewer response for version 1

**DOI:** 10.21956/openreseurope.21752.r54542

**Published:** 2025-06-14

**Authors:** Joseph Nwadiuko

**Affiliations:** 1 University of Pennsylvania, Philadelphia, Philadelphia, USA

This is a good and very timely article. Comparative research of conditions in immigration detention is rare, so I find this a valuable step and hope that more analyses like these are forthcoming.

*Introduction*

 1) As stated above, I think that the contribution of comparative approaches is important. I would make that gap a bit more clear, and include certain studies that have at least tried to conduct comparative research, although not quite with the aims or countries that your team has in mind, such as here: ( refer to 1 and 2 ) Why does your analysis focus on Spain and Italy in particular? Is this due to Spain and Italy receiving more immigrants than other places, or having more concerns about abuses in those countries?

 3) Your team mentions that “these centres have become deportation devices and detention centres and are present at all stages of migration control”. I’m not certain what “stages of migration control” refers to explicitly here. Please clarify.

 4) You mention the phrase “irregular situation” twice. This is term that doesn’t translate well in English so a definition would be helpful. (You might mean “irregular status”, but I’m not sure).

*The Spanish CIE and the Italian CPR: a comparative overview*

 5) In some countries (e.g., US and I believe Australia), immigration detention facilities are heavily privatized. Is that a reality in these two countries?

 6) The term “judge of control” also doesn’t quite translate well in English (and might be a position unique to Spain), I would recommend using the explicit Spanish term (juez de control, I presume), and explain it a bit better in a couple words (e.g., “a CIE site-specific court magistrate who monitors conditions and ensures detainee rights are upheld”—not sure if that’s precisely it, just setting it up as an example). For context, for the US and other settings, immigration judges don’t rule on conditions cases—that goes to the same federal courts as any non-immigration litigation would.

 7) You state:” For both countries, the effective repatriations amount to slightly more than 50% of the total inmates and have remained constant over the years.” Please cite this statement and provide a precise figure for both countries.

*Results*

 8) I’d strongly recommend having a Table 3 with results, broken down by country and codes as you have it in the text. You might even combine it with Table 2 if needed for efficiency.

 9) Explain “emergency doors”. Are these doors for exits, or for entry to emergency care places?

If applicable, is the statistical analysis and its interpretation appropriate?

Yes

Is the study design appropriate and is the work technically sound?

Yes

Is the work clearly and accurately presented and does it engage with the current literature?

Yes

Are the conclusions drawn adequately supported by the results?

Yes

Are sufficient details of methods and analysis provided to allow replication by others?

Partly

Are all the source data and materials underlying the results available?

Yes

Reviewer Expertise:

Health Policy, Immigration Detention

I confirm that I have read this submission and believe that I have an appropriate level of expertise to confirm that it is of an acceptable scientific standard, however I have significant reservations, as outlined above.

## References

[1] : The Detention of Migrant Children: A Comparative Study of the United States and Mexico. *International Journal of Refugee Law* .2020;32(2) : 10.1093/ijrl/eeaa014 227-253 10.1093/ijrl/eeaa014

[2] globaldetentionproject.org/wp-content/uploads/2018/10/Harm-Reduction-in-Immigration-Detention-GDP-Norwegian-Red-Cross.pdf.

# Reviewer report: Reviewer response for version 1

**DOI:** 10.21956/openreseurope.21752.r54214

**Published:** 2025-05-27

**Authors:** M. Alvi Syahrin

**Affiliations:** 1 Immigration Polytechnic University in Depok, Jawa Barat, Jawa Barat, Indonesia

**Introduction and Relevance:**

 This article addresses the complex and timely topic of immigration detention centres in Spain and Italy by conducting a comparative study between 2018 and 2023. Given the increasing relevance of detention as a border control mechanism in Europe, and the heightened scrutiny of the European Union's migration policies under the 2023 New Pact on Migration and Asylum, this work is of significant scientific and political importance. The paper is especially relevant as it focuses on two countries—Spain and Italy—that serve as principal gateways into Europe for migrants and asylum seekers. By drawing attention to structural deficiencies and systemic rights violations, the authors place a necessary spotlight on areas that often lack public transparency.

 The introduction contextualizes detention centres within EU policy, legal frameworks, and institutional practices. However, the article could benefit from a more pronounced articulation of the central research questions and hypothesis. While the general aim—to compare systemic and legal deficiencies in detention—is clearly stated, it would be advantageous to explicitly pose these in the form of testable or investigable research questions to align with qualitative research best practices.

**Methodological Rigor and Approach**

 The article adopts a qualitative content analysis approach, drawing on 16 official reports (10 Italian and 6 Spanish) produced by the Spanish Ombudsman and the Italian National Guarantor between 2018 and 2023. The use of NVivo 14 software for data organization and thematic coding ensures methodological rigor in data management. The justification for report selection—focusing on comparability and omitting non-equivalent formats—is valid and well-explained.

 Three core thematic blocks guide the analysis: (1) Structural and operational conditions, (2) Detainee rights, and (3) Adequacy of healthcare, legal, and social services. This tripartite framework is logical and aligns with the central objectives of the study. Coding categories were created a priori and adjusted inductively based on document content, which is consistent with accepted practices in qualitative research.

 Nonetheless, the study could be strengthened through the following improvements:

- **Transparency in Coding Procedures:** The article lacks detailed discussion on intercoder reliability or procedures undertaken to ensure consistency in thematic interpretation.
- **Triangulation:** Although the authors rely exclusively on institutional reports, adding triangulation with media accounts, NGO reports, or first-hand interviews would enhance the trustworthiness of findings.
- **Language Translation:** The authors note limitations related to report translation but do not describe steps taken to mitigate potential misinterpretations or biases.

**Analysis of Results**

 The findings confirm that both Spain and Italy suffer from deeply entrenched issues in their detention systems. The structural conditions in both countries are described as outdated, unsanitary, and often prison-like. In Spain, examples include surveillance deficits, mixed-use spaces that fail to ensure detainee privacy, and heating failures. In Italy, there are reports of insect infestations, lack of furnishings, and deteriorating hygiene conditions. The comparative dimension is particularly valuable: for instance, the longer maximum detention duration in Italy (180 days) vs. Spain (60 days) is explored as both a symptom and enabler of systemic issues.

 Detainee rights are critically undermined across both systems. In Spain, access to communication and legal counsel is limited and inconsistently applied. Italy presents even more severe barriers, including limited lawyer access, delays in asylum procedures, and mobile phone restrictions. The analysis is supported by specific examples from both countries, adding to the credibility of claims.

 A major strength of the article lies in its detailed treatment of healthcare conditions. Reports from both countries document absent or untrained medical staff, a lack of psychiatric care, and overprescription of sedatives. These findings are significant and align with broader literature on the health consequences of immigration detention, which link such conditions to PTSD, depression, and long-term trauma.

**Discussion and Theoretical Contribution**

 The discussion situates the findings within broader academic and policy discourses on detention and securitization. The authors use the concept of securitization to critique the framing of migrants as threats, which justifies extended detention and punitive environments. This conceptual lens is powerful and aligns with critical migration literature. Moreover, the discussion incorporates the Nelson Mandela Rules and other international human rights standards to highlight the legal deficits in the operation of CIE and CPR.

 However, the article could benefit from deeper engagement with sociological and legal theories of institutional governance, such as Goffman’s theory of total institutions or Agamben’s notion of the “state of exception.” Such theoretical anchors would enable a richer understanding of how institutional practices both reflect and reinforce structural violence.

 Additionally, while the authors mention the inadequacies of administrative detention as a migration control tool, the argument would be stronger with more data on deportation effectiveness, asylum approval rates, or cost-efficiency comparisons.

**Scientific Contribution and Originality**

 This study provides an original and much-needed contribution to comparative studies in European migration governance. It moves beyond surface-level descriptions by using institutional data systematically analyzed over time. The focus on the operational and rights-based consequences of detention adds value to policy and academic discussions.

 The originality lies in its specific comparative framework, the incorporation of national-level oversight data, and the temporal coverage of five years. While studies exist on CIE or CPR separately, few offer this level of comparative detail grounded in official documentation.

**Limitations**

 Despite its strengths, the study has limitations:

1. **Lack of Primary Data**: The absence of interviews with detainees or staff limits the perspective to institutional viewpoints.
2. **Translation Bias**: The authors acknowledge the risk of distortion in translating institutional documents but provide no evidence of linguistic mitigation strategies.
3. **Temporal Constraints**: Although the study covers 2018–2023, the most recent effects of the 2023 New EU Pact on Migration and Asylum are still unfolding and thus not fully analyzed.
4. **Sample Asymmetry**: The larger number of Italian reports (10 vs. 6) may have skewed comparative insights, although the authors attempted normalization through selective coding.

**Policy and Practical Implications**

 The article offers clear and well-founded recommendations, including:

- Structural investment in facility upgrades
- The appointment of qualified healthcare personnel, especially in mental health
- The establishment of standardized legal assistance and complaint mechanisms
- The transition toward non-custodial alternatives such as community-based care and case management

These recommendations are aligned with international human rights standards and supported by both data and literature. Importantly, the article underscores that detention does not fulfill its intended purpose (migration control), thus suggesting inefficiency as well as inhumanity.

**Conclusion**

 This article makes a compelling case for the urgent reform of detention centres in Spain and Italy. Through its systematic, data-driven, and rights-oriented approach, it provides evidence that current practices violate both national and international standards. The use of qualitative content analysis and official reports gives the findings legitimacy, while the comparative dimension allows for nuanced insights into systemic challenges.

 Further research should include detainee perspectives, institutional ethnographies, and broader EU-wide comparisons to expand the knowledge base. Nonetheless, this article stands as a strong, policy-relevant contribution to debates on detention, migration governance, and human rights compliance in Southern Europe.

If applicable, is the statistical analysis and its interpretation appropriate?

Yes

Is the study design appropriate and is the work technically sound?

Yes

Is the work clearly and accurately presented and does it engage with the current literature?

Yes

Are the conclusions drawn adequately supported by the results?

Yes

Are sufficient details of methods and analysis provided to allow replication by others?

Yes

Are all the source data and materials underlying the results available?

Yes

Reviewer Expertise:

migration, refugees, detention

I confirm that I have read this submission and believe that I have an appropriate level of expertise to confirm that it is of an acceptable scientific standard.
